# Genetic Characterization of 191 Probands with Inherited Retinal Dystrophy by Targeted NGS Analysis

**DOI:** 10.3390/genes15060766

**Published:** 2024-06-12

**Authors:** Alessandra Mihalich, Gabriella Cammarata, Gemma Tremolada, Emanuela Manfredini, Stefania Bianchi Marzoli, Anna Maria Di Blasio

**Affiliations:** 1 Molecular Biology Laboratory, Istituto Auxologico Italiano IRCCS, 20145 Milan, Italy; e.manfredini@auxologico.it (E.M.); a.diblasio@auxologico.it (A.M.D.B.); 2 Neuro-Ophthalmology Center and Electrophysiology Laboratory, Department of Ophthalmology, Istituto Auxologico Italiano IRCCS, 20145 Milan, Italy; g.cammarata@auxologico.it (G.C.); g.tremolada@auxologico.it (G.T.); s.bianchimarzoli@auxologico.it (S.B.M.)

**Keywords:** NGS, IRDs, genetic diagnosis

## Abstract

Inherited retinal diseases (IRDs) represent a frequent cause of blindness in children and adults. As a consequence of the phenotype and genotype heterogeneity of the disease, it is difficult to have a specific diagnosis without molecular testing. To date, over 340 genes and loci have been associated with IRDs. We present the molecular finding of 191 individuals with IRD, analyzed by targeted next-generation sequencing (NGS). For 67 of them, we performed a family segregation study, considering a total of 126 relatives. A total of 359 variants were identified, 44 of which were novel. Genetic diagnostic yield was 41%. However, after stratifying the patients according to their clinical suspicion, diagnostic yield was higher for well-characterized diseases such as Stargardt disease (STGD), at 65%, and for congenital stationary night blindness 2 (CSNB2), at 64%. Diagnostic yield was higher in the patient group where family segregation analysis was possible (68%) and it was higher in younger (55%) than in older patients (33%). The results of this analysis demonstrated that targeted NGS is an effective method for establishing a molecular genetic diagnosis of IRDs. Furthermore, this study underlines the importance of segregation studies to understand the role of genetic variants with unknow pathogenic role.

## 1. Introduction

Inherited retinal diseases (IRDs) constitute a group of diseases with vast clinical and genetic heterogeneity affecting about 1 in 2000 to 3000 individuals [1]. They are characterized by the progressive deterioration or early loss of photoreceptors and other neuronal and glial cells [2]. According to the Retinal Information Network (RetNet, 2023) [3] more than 300 genes, many of which may cause more than one phenotype, have been implicated as causes of IRDs. These genes can be involved in a variety of processes and functions [4] and their tissue expression can range from exclusive expression in the retina to ubiquitous expression across the body (Genotype-Tissue Expression GTEx https://gtexportal.org accessed on 9 May 2024).

The different forms of IRD may present with considerable clinical overlap. This often precludes the assessment of a diagnosis on the basis of the disease phenotype alone [5]. Genetic diagnosis has improved the knowledge of these heterogeneous conditions and the introduction of next-generation sequencing (NGS) has further improved molecular genetic diagnosis of inherited retinal diseases. In certain clinically well-defined phenotypes of IRDs, such as retinoschisis, the disease is linked to variations in one specific “disease gene” (RS1) and the genetic diagnostic rate can exceed 90% [6]. In contrast, in other subtype of IRDs, like retinitis pigmentosa (RP), an extreme level of genetic heterogeneity is observed [7] and in patients affected by RP, variants localized in more than 100 “disease genes” have been reported [8]. Further complexities are provided by those genes whose variants are associated with multiple IRD phenotypes or with multiple inheritance patterns of the same IRD phenotype.

As a consequence, more information is needed to better describe the complex landscape of IRDs.

In this study, we present a genetic investigation of a cohort of 191 probands resident in Italy and clinically diagnosed or suspected with IRD. In 67 cases, samples from additional family members were analyzed to verify segregation of the sequence variants identified in the index patient and to understand their pathogenic role. The results obtained will provide new information about genotype–phenotype correlations of novel variants.

## 2. Materials and Methods

### 2.1. Ethical Approval

The institutional review board approved the study, which is in adherence with the Declaration of Helsinki. All patients underwent genetic counseling and signed informed consent.

### 2.2. Patients and Clinical Evaluation

In this retrospective study, we gathered data from 191 patients (107 male, 56%; 84 female, 44%) clinically suspected of having an inherited retinal disease based on the evaluation of available clinical records. The patients were divided into 11 groups according to their clinical diagnosis. The group defined as “rod–cone dystrophy” (RCD) included patients with an electroretinogram (ERG) pattern suggesting a prevalent rod-system dysfunction. The group defined as “retinitis pigmentosa” (RT) included patients with rod–cone dystrophies with the classic phenotype of retinal pigmentary changes in the form of bone-spicule and pigment clumping.

One hundred and sixteen patients underwent examination at the Neuro-Ophthalmology and Electrophysiology Service of the Istituto Auxologico Italiano and received a comprehensive neuro-ophthalmological examination, as previously described [9]. Seventy-five patients, with a clinical diagnosis made in another center, were assessed in our laboratory for genetic analysis. Clinical records were available for 52 of them, while for 23 patients, only medical prescriptions were available.

In 67 cases, samples from family members (totaling 126 individuals: 85 parents, 19 siblings, 14 sons, 2 aunts, 2 uncles, 2 cousins, 1 grandfather and 1 nephew) were utilized to study the segregation of the sequence variants identified in the index patient and to explore their role (Table 1).

### 2.3. Genetic Testing

Patients collected from September 2014 to March 2021 were tested via targeted NGS, using a panel composed of 32 selected genes associated with inherited retinal disorders in the RetNet database, the OMIM (Online Mendelian Inheritance in Man) and a literature search (Table S1, Panel 1). From April 2021 to June 2023 patients were analyzed using an updated panel of 63 genes (Table S1, Panel 2). All selected genes were present in Orphanet and were associated with an inherited retinal disorder and each gene in the Human Gene Mutation Database (HGMD) had more than 20 registered variants. 

Genes were classified according to their known inheritance (as reported in the HGMD) as autosomal recessive (AR), autosomal dominant (AD), or X-linked (XL) and autosomal dominant or recessive (AD/AR).

### 2.4. Mutation Screening

Peripheral blood samples were obtained from patients and available family members. Genomic DNA was extracted, as described previously [9]. NGS libraries were prepared according to the manufacturer’s instructions (NGS Custom Panel Enrichment Library Preparation, Illumina, San Diego, CA, USA). Illumina NGS Custom Panel Enrichment was designed, as already described [8]. The total coverage of the target genes by the designed amplicons was >95% and the mean depth of coverage was 1130X. NGS was performed on a NextSeq 500 sequencer (Illumina, San Diego, CA, USA).

### 2.5. Bioinformatics Data Analysis

Data collected from NGS experiments were analyzed using an in-house bioinformatic pipeline, as already described [10].

### 2.6. Annotation and Classification of Genetic Variants

The genetic variants were annotated using the software Annovar [11,12] and then were classified into five categories: pathogenic (P) (class 5), likely-pathogenic (LP) (class 4), variant of uncertain significance (VUS) (class 3), likely benign (LB) (class 2) and benign (B) (class 1), according to the ACMG guidelines [13] using the classification tools Franklin (Genoox) or Varsome. Then, every single variant was annotated using ALAMUT^®^ VISUAL (Interactive BioSoftware, Rouen, France) and the following databases: HGMD Professional, ClinVar and LOVD3. A variant was classified as “novel” when not present in the archives mentioned above. The impact of novel splice-site variants was assessed using specific splice-site prediction programs present in ALAMUT^®^ VISUAL (SpliceSiteFinder-like, MaxEntScan, GeneSplicer, NNSPLICE and Human Splicing Finder). 

### 2.7. Mutation Validation

All the genetic variants were confirmed by Sanger sequencing.

### 2.8. Multiplex Ligation-Dependent Amplification (MLPA)

The presence of deletions or duplications in genes *ABCA4*, *EYS* and *USH2A* was investigated by MLPA using kits P151 and P152 (*ABCA4*), P328-A3 (*EYS*), P361 and P362 (*USH2A*), following the manufacturer’s instructions (MRC-Holland, Amsterdam, The Netherlands). Fragment size separation was conducted on the Applied Biosystems Inc. (ABI) 3500 DNA analyzer with POP7 polymer capillary electrophoresis. Data analysis was performed using Coffalyser MLPA analysis software.

### 2.9. Analysis of ABCA4 Selected Deep-Intronic Variants

To test the occurrence of twelve intronic *ABCA4* variants (c.570+1798A>G, c.769-784C>T, c.769-788A>T, c.859-506G>C, c.4539+1100A>G, c.4539+1106C>T, c.4539+2001G>A, c.4539+2028C>T, c.4539+2064C>T, c.4539+2065C>G, c.5196+1056A>G and c.5196+1137G>A) in 23 patients that carried only one pathogenic or probably pathogenic variant in ABCA4, we designed primers in intron 5, 6, 7, 30 and 36 to perform PCR and Sanger sequencing.

### 2.10. Genetic-Finding Interpretation

For classification of cases we referred to Weisschuh et al. and Falsini et al. [14,15]. In brief, a patient case was considered “solved” if:

(1) The patient harbored a variant classified as “pathogenic” or “likely pathogenic” according to the ACMG classification and/or documented in the literature (ClinVar, HGMD Professional, LOVD3) within a gene recognized to cause an autosomal dominant disease. Alternatively, for male patients, a gene associated with an X-linked disease was considered.
(2) The patient harbored two variants (suspected or shown to lie on separate alleles) classified as “pathogenic” or “likely pathogenic” for the ACMG classification and/or in literature (ClinVar, HGMD Professional or LOVD3), in a gene known to cause an autosomal recessive disease.
(3) The patient had a VUS variant on a dominant gene or, if the patient was a male, on an X-linked gene with the support of the family segregation study.

A patient case was considered “possibly solved” if:

(1) The patient harbored, in a gene known to cause an autosomal recessive disease, on one allele, a missense variant reported as “pathogenic” in literature (ClinVar, HGMD Professional or LOVD3) or for the ACMG classification and on the other allele a novel splice-site variant predicted to be pathogenic by “in silico” studies.
(2) The patient harbored two variants, one classified as “pathogenic” or “likely pathogenic” and the other classified as VUS, in a gene known to cause an autosomal recessive disease.

A patient case was considered “inconclusive” if the patient harbored one variant classified as VUS in a dominant gene without the support of the family segregation study, or two variants classified as VUS in recessive genes. These cases needed other studies. 

Finally, a patient case was considered “unsolved” if the patient did not have a variant explaining the phenotype or if they harbored only one variant in a recessive gene.

## 3. Results

### 3.1. Patient Characteristics

**Figure 1:**
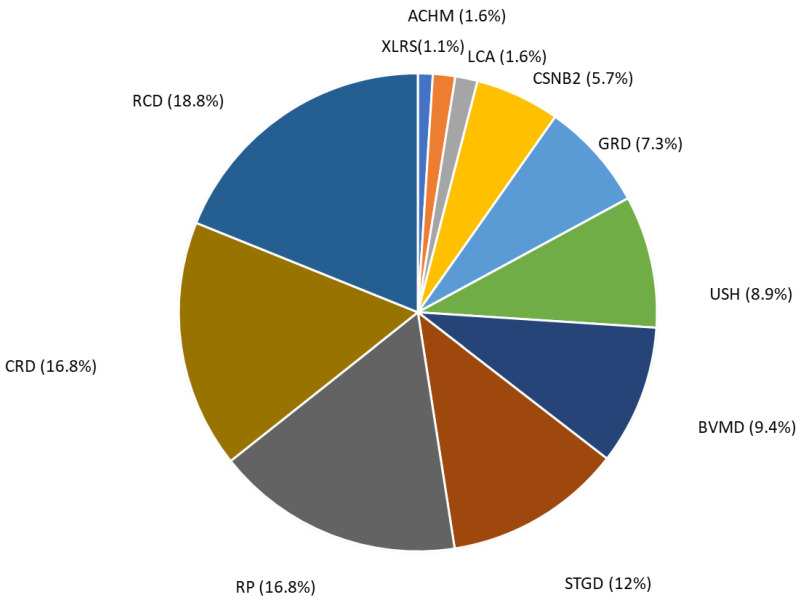
Distribution of patients according to their clinical diagnosis. “rod–cone dystrophy” (RCD), “cone and cone–rod dystrophy” (CRD), “retinitis pigmentosa” (RT), “Stargardt disease” (STGD), “best vitelliform macular dystrophy” (BVMD), “Usher syndrome” (USH), “generalized retinal dystrophy” (GRD), “congenital stationary night blindness 2” (CSNB2), “Leber congenital amaurosis” (LCA), “achromatopsia” (ACHM) and “retinoschisis” (XLRS).

We performed diagnostic NGS testing in 191 patients referred with clinical indications of IRD based on evaluation of available clinical records (family history, ophthalmologist evaluation, perimetry, retinal multimaging and electroretinograms (ERGs)) (Table S2). The patients were divided into 11 groups according to their clinical diagnosis (Figure 1). The group “rod–cone dystrophy” (RCD) represented the most common clinical diagnosis (18.8%), followed by “cone and cone–rod dystrophy” (CRD) and “retinitis pigmentosa” (RT) (16.8%), “Stargardt disease” (STGD) (12%), “best vitelliform macular dystrophy” (BVMD) (9.4%), “Usher syndrome” (USH) (8.9%), “generalized retinal dystrophy” (GRD) (7.3%), “congenital stationary night blindness 2” (CSNB2) (5.7%), “Leber congenital amaurosis” (LCA) (1.6%), “achromatopsia” (ACHM) (1.6%) and “retinoschisis” (RS1) (1.1%) (Figure 1).

In 67 cases (Tables S2 and S5) other family members were analyzed for segregation studies for a total of 127 family members.

### 3.2. Genetic Diagnosis

**Figure 2:**
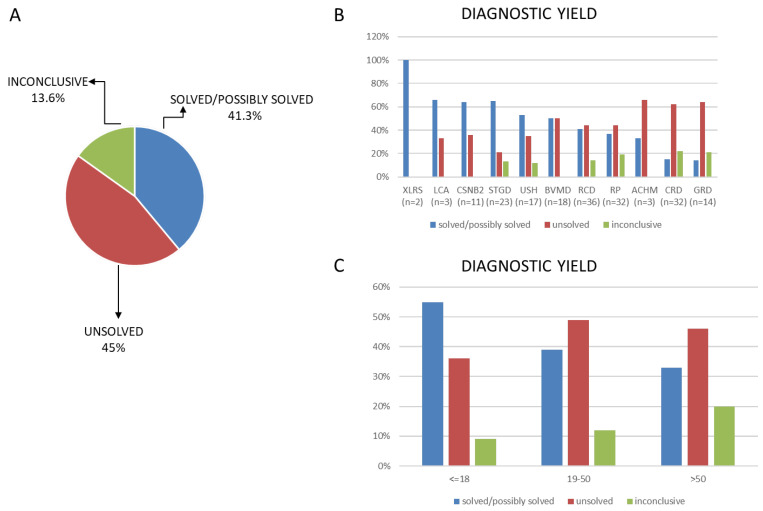
Genetic diagnosis results: (**A**) diagnostic yield in total cohort; (**B**) diagnostic yield according to the disease type “rod–cone dystrophy” (RCD), “cone and cone–rod dystrophy” (CRD), “retinitis pigmentosa” (RT), “Stargardt disease” (STGD), “best vitelliform macular dystrophy” (BVMD), “Usher syndrome” (USH) (8.9%), “generalized retinal dystrophy” (GRD), “congenital stationary night blindness 2” (CSNB2), “Leber congenital amaurosis” (LCA), “achromatopsia” (ACHM) and “retinoschisis” (XLRS) and (**C**) diagnostic yield according to the patients’ ages.

Seventy-nine (41.3%) cases were considered “solved” or “possibly solved” as a plausible molecular genetic diagnosis could be established. Among them, in 46 patients the diagnosis was confirmed by segregation analysis in other family members. Twenty-six (13.6%) cases were “inconclusive” as it was not possible to determine if the genetic variants found were responsible of the disease. Eighty-six (45%) cases were considered “unsolved” as no plausible molecular genetic diagnosis was established (Figure 2A). Diagnostic yield varied according to the disease type (Figure 2B). It was higher for clinically well-defined forms of IRD, such as retinoschisis (two out of two patients, 100%), LCA (two out of three patients, 66%), STGD (65%) and CSNB2 (64%). It was lower in patients with generic diagnosis of generalized retinal dystrophy GRD (14%). Diagnostic yield was higher in the patient group where family segregation analysis was possible (68%) and was higher in younger patients (55%) (<=18) compare with older ones (33%) (Figure 2C).

### 3.3. Genetic Findings

Potential disease-causing variants (classified as VUS, LP or P) were found in 166 (86.9%) individuals (Table S3). In 94 (49.2%) cases we found variants in more than one gene. Genotypes of all probands are listed in Table S3. Table S4 contains variants’ complete description. 

For genes *ABCA4*, *EYS* and *USH2A*, multiplex ligation-dependent probe amplification (MLPA) was performed in order to detect possible deletions or duplications in the 45 patients that were found to carry only a single variant in these genes after NGS analysis. Four deletions were detected by MLPA, two in EYS (cases 100 and 132) and two in USH2A (cases 39 and 110) (Table S3); we did not define breakpoints for these large deletions.

A total of 361 variants were reported (Table S4). Of these, 44 were novel at the time of manuscript submission (Table S4). The following genes (Panel 2) did not harbor any disease-causing variant in our proband cohort: *ARL6*, *CDHR1*, *CFAP410*, *CLRN1*, *FAM161A*, *GDF6*, *IQCB1*, *LCA5*, *LRAT*, *NMNAT1*, *PDE6C*, *PROM1*, *RDH12*, *RIMS1* and *WHRN*.

In 21 (11%) patients one genetic variant in genes with X-linked inheritance was detected. Sixty-seven (35%) patients presented one or more genetic variants in genes with autosomal dominant inheritance or in genes with inheritance that could be autosomal dominant or autosomal recessive. In 50 patients (26%), two genetic variants in one or more genes with autosomal recessive inheritance were found. In 42 patients (22%) only one genetic variant in one or more genes with autosomal recessive inheritance was detected. 

A total of 98 pathogenic or likely pathogenic variants were found in 25 genes: 42 missense variants (42.8%), 24 deletions (24.4%), 12 nonsense variants (12.2%), 12 splice-site variants (12.2%) and 8 duplications (8.1%).

**Figure 3:**
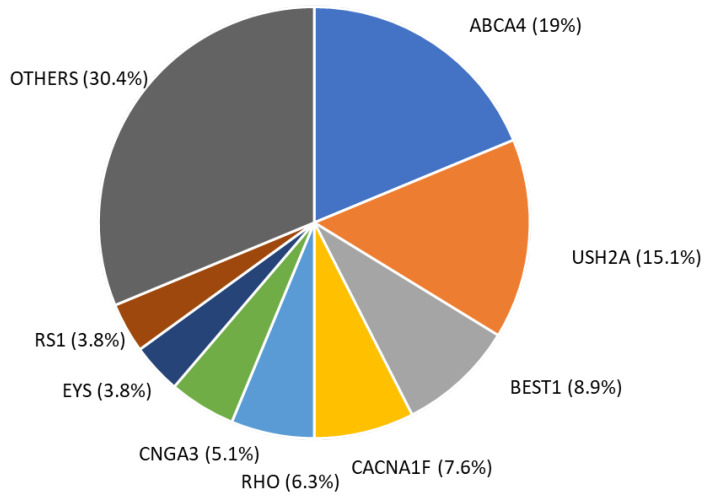
Distribution of patients with genetic diagnosis according to the gene where variants were localized.

*ABCA4* was the most common disease-causing gene in our cohort and two pathogenic variants were found in 15 patients (7.8%), 13 of them affected by Stargardt disease, 1 with cone-dystrophy and 1 with Usher syndrome. The second disease-causing gene was *USH2A*, with 12 patients with two variants (6.2%), 5 patients affected by Usher syndrome, 4 with rod-cone dystrophy, 2 with retinitis pigmentosa and 1 with CSNB2. Variants in *BEST1* were responsible for 7 cases of best vitelliform macular dystrophy (3.6%). *CACNA1F* (X-linked) variants were found in six male patients (3.1%), five with CSNB2 and one with retinoschisis. Mutations in *CNGA3* caused cone dystrophy in three patients and achromatopsia in one patient (2%). Variants in RHO were responsible for retinitis pigmentosa in three patients, rod–cone dystrophy in one patient and retinal dystrophy in one case (2.6%). Variants in other genes were found in 25 patients (13%) (Figure 3).

A total of 45 different variants of the *ABCA4* gene were present in our population. The more representative variant was c.5882G>A p.(Gly1961Glu) (eight patients) then c.982G>T p.(Glu328*) and c.2588G>C p.(Gly863Ala) (two patients each). Three variants were novel at the time of manuscript submission and two of these were in trans with c.5882G>A. Twenty-three probands with one heterozygous *ABCA4* allele were analyzed for the presence of twelve deep-intronic variants that were reported as the more frequent in the population [16] (c.570+1798A>G, c.769-784C>T, c.769-788A>T, c.859-506G>C, c.4539+1100A>G, c.4539+1106C>T, c.4539+2001G>A, c.4539+2028C>T, c.4539+2064, c.4539+2065C>G, c.5196+1056A>G and c.5196+1137G>A) but none was identified. 

Fifty-four different variants in the *USH2A* gene were present in our patients; the more representative variants were c.11927C>Tp.(Thr3976Met) (three patients) and c.2276G>T p.(Cys759Phe) (two patients) and nine variants were novel. 

### 3.4. Segregation Studies

Segregation studies were conducted in family members (126) of 67 patients (Table S5), as described above, and the genetic diagnosis rate was of 68.6%. The segregation study was informative in 58 cases (88%). In 28 cases, we could ascertain the presence of two variants in two different alleles, in 11 cases we were able to confirm the variant’s segregation with the phenotype, and in 2 cases (63–100) there were too many variants in different genes with AR heritability to determine which gene was responsible for the phenotype. In 15 cases, family studies suggested that the candidate variant was unlikely to cause the disease (patients 35, 42, 77, 103, 105, 117, 129, 134, 136, 140, 144, 154, 173, 175 and 177). 

## 4. Discussion

For this report, we retrospectively analyzed the clinical and genetic data of 191 patients affected by inherited retinal disease, and their family members (total 317 patients), referred to our Institution from September 2014 to August 2023. Molecular testing was performed with a panel composed of 32 selected genes that, from April 2021, was integrated with an additional 32 genes. Furthermore, in selected cases, MLPA for genomic rearrangements of genes *ABCA4*, *EYS* and *USH2A* was also performed. 

The results of this study demonstrate a genetic diagnosis detection rate of 41%, which is relatively low compared to that of other studies. Indeed, the meta-analysis conducted by Britten-Jones et al. on 105 publications [17], as well as other studies [15,16,17,18,19,20], reported a median diagnostic yield of 61.3%. This difference is most likely due to the very stringent parameters that we used to consider a case “solved”. Indeed, if less stringent parameters had been used, cases that we defined as “inconclusive” (13.6%), could have been classified as “possibly solved” and our detection rate would have increased to 54%.

Moreover, among the 58 cases analyzed with the second panel, only 31% were solved and 27% were inconclusive. This result is due to the finding of many variants localized in genes at present not well studied that, as a consequence, have unknown pathogenetic significance.

The diagnostic yield varied according to the disease type. It was highest for clinically well-defined forms of IRD such as retinoschisis (two out of two patients (100%)), LCA (66%), STGD (65%) and CSNB2 (64%) and was lower in patients with generic diagnosis of retinal dystrophy GRD (14%). These results agree with those of Britten-Jones et al. who registered higher diagnostic rates in IRDs with less genetically heterogenous phenotypes like retinoschisis and CSNB2 [17].

In the population studied, we could identify putative disease-causing variants in 55% of patients younger than 18 years, while the detection rate drastically cd to 33% in the group of patients older than 50 years. This result confirms data reported by Shanks et al. and suggests that the diagnostic yield in patients with early onset disease is higher than that in those with later onset disease, probably because the latter may have a multigenic or multifactorial etiology [21]. 

In the group of patients where other family members were studied, the detection rate was 68.6%. The remaining 31.4% of patients without genetic diagnosis were either unsolved (23.8%) or inconclusive (7.4%). This result underscores the importance of segregation studies not only in understanding the role of genetic variants with unknown pathogenic significance. For example, in four cases (79, 99, 135 and 166) which were classified as “inconclusive”), each of which had two variants of the same gene, the segregation study would have allowed us to determine whether they were on the same allele or not. In three cases, two with variants in genes with autosomal dominant inheritance (cases 43 and 148) and one with a variant in a gene with X-linked inheritance (case 158, male), the segregation study would have allowed us to determine the pathogenicity of the identified variants. In ten other cases (111, 112, 113, 114, 115, 142, 162, 167, 169 and 186), each with multiple identified variants, family studies may have helped us to understand the role of each individual variant.

The variability of clinical features among patients carrying variants in the same gene is sometimes remarkable and could depend on a number of other genetic determinants. In our cohort, fifteen patients have sequence variations in two or more genes that potentially could have been the cause of IRD. Among these, in three cases genetic analysis could explain the complex situation. Patient 80 presented a variant in *RS1* gene that was likely pathogenic and probably responsible for the patient’s retinoschisis. However, genetic analysis also indicated the presence of a *CACNA1F* variant that could have been responsible for the patient’s night blindness. In patient 149, with retinal dystrophy and night blindness, genetic analysis suggested that the two variants in *AIPL1* gene, in two different alleles, could have been responsible for patient’s retinal dystrophy. The presence in the proband, and in his mother, of a *TRPM1* variant, a gene associated with night blindness, induced us to reevaluate the mother’s clinical phenotype and this led to the discovery that she also had difficulties with night vision (the patient is currently waiting for ERG analysis). In patient 150, the *RHO* variant was most likely the cause of the retinitis pigmentosa but a variant in the *TRPM1* gene could have influenced the phenotype.

In four cases the first generic clinical diagnosis was switched to a best-defined syndrome after the results of the genetic analysis. In three cases clinical diagnosis was rod–cone dystrophy (pat 65 and 81) or retinal dystrophy (pat 123) and the genetic analysis demonstrated two mutations in the *USH2A* gene in different alleles. Additional clinical evaluation revealed, in patients 65 and 81, mild hearing impairment consistent with Usher syndrome. In patient 104, with clinical presentation of rod–cone dystrophy, genetic results indicated a pathogenic variant in the RS1 gene, suggesting a diagnosis of retinoschisis. 

## 5. Conclusions

In conclusion, the results reported herein pave the way to further classification of variants and highlight the importance of phenotype–genotype correlation studies. Nowadays, a large number of potential therapeutic approaches, such as gene replacement, gene silencing, antisense oligonucleotides and genome editing are available and were recently demonstrated to be effective [22]. Indeed, a genetic diagnosis is of utmost importance for predicting familial risk and disease progression and for eligibility for clinical trials or pharmacological treatments. 

## Figures and Tables

**Table 1:** General characteristics of the population studied.

	Total	Segregation Study
Patients	191	67 (35%)
Male	107 (56%)	38 (20%)
Female	84 (44%)	29 (15%)

## Data Availability

Raw data are available, upon request, in Zenodo Repository.

## Supplementary Materials

The following supporting information can be downloaded at: https://www.mdpi.com/article/10.3390/genes15060766/s1, Table S1: Genes in the panels. Table S2: Patients with their clinical diagnosis and genetic diagnosis outcome. Table S3: Genetic analysis results for each patient. Table S4: Genetic variants identified. Table S5: Genetic analysis results of patients’ relatives.
