# Supplementary material for: Genetic Characterization of 191 Probands with Inherited Retinal Dystrophy by Targeted NGS Analysis
Source: Genes (Basel). 2024 Jun 12;15(6):766. doi: 10.3390/genes15060766 (PMC11203276; doi:10.3390/genes15060766)
Supplement: Supplementary file 1 [file genes-15-00766-s001.zip › TABLE CAPTION.pdf]

TABLE CAPTION

Table S1: Genes present in Panel 2 and in column E genes already present in Panel 1; Table S2: Patients analyzed, their clinical diagnosis, segregation studies; Table S3: Genotype of all probands; Table S4: List of variants; Table S5: Genotype of all relatives, the number refers to the probands ID.
